# Behavioural and environmental risk factors associated with primary schoolchildren’s overweight and obesity in urban Indonesia

**DOI:** 10.1017/S1368980023000897

**Published:** 2023-08

**Authors:** Margarita de Vries Mecheva, Matthias Rieger, Robert Sparrow, Erfi Prafiantini, Rina Agustina

**Affiliations:** 1 The International Institute of Social Studies of Erasmus University Rotterdam, 2518 AX The Hague, the Netherlands; 2 Development Economics Group, Wageningen University, Wageningen, the Netherlands; 3 Department of Nutrition, Faculty of Medicine, Universitas Indonesia – Dr Cipto Mangunkusumo General Hospital, Jakarta, Indonesia; 4 Human Nutrition Research Center, Indonesian Medical Education and Research Institute (HNRC-IMERI), Faculty of Medicine, Universitas Indonesia, Jakarta, Indonesia

**Keywords:** Childhood overweight and obesity, Risk factors, Home food environment, Indonesia

## Abstract

**Objectives:**

To aid the design of nutrition interventions in low- and middle-income countries undergoing a nutrition transition, this study examined behavioural and environmental risk factors associated with childhood overweight and obesity in urban Indonesia.

**Design:**

Body height and weight of children were measured to determine BMI-for-age Z-scores and childhood overweight and obesity status. A self-administered parental survey measured socio-economic background, children’s diet, physical activity, screen time and parental practices. Logistic and quantile regression models were used to assess the association between risk factors and the BMI-for-age Z-score distribution.

**Setting:**

Public primary schools in Central Jakarta, sampled at random.

**Participants:**

Children (*n* 1674) aged 6–13 years from 18 public primary schools.

**Results:**

Among the children, 31·0 % were overweight or obese. The prevalence of obesity was higher in boys (21·0 %) than in girls (12·0 %). Male sex and height (aOR = 1·67; 95 % CI 1·30, 2·14 and aOR = 1·16; 95 % CI 1·14, 1·18, respectively) increased the odds of being overweight or obese, while the odds reduced with every year of age (aOR = 0·43; 95 % CI 0·37, 0·50). Maternal education was positively associated with children’s BMI at the median of the Z-score distribution (*P* = 0·026). Dietary and physical activity risk scores were not associated with children’s BMI at any quantile. The obesogenic home food environment score was significantly and positively associated with the BMI-for-age Z-score at the 75th and 90th percentiles (*P* = 0·022 and 0·023, respectively).

**Conclusions:**

This study illustrated the demographic, behavioural and environmental risk factors for overweight and obesity among primary schoolchildren in a middle-income country. To foster healthy behaviours in primary schoolchildren, parents need to ensure a positive home food environment. Future sex-responsive interventions should involve both parents and children, promote healthy diets and physical activity and improve food environments in homes and schools.

The increasing prevalence of overweight and obesity in low- and middle-income countries (LMIC) has been linked to a nutritional transition towards increased consumption of energy-dense processed foods that is correlated with decreased levels of people’s physical activity and various environmental factors^(1)^. These changes in energy intake and expenditure were also observed in children and may explain the rapid increase in childhood overweight and obesity in many regions^(2)^.

The setting of this study, urban Indonesia, reflects general trends in LMIC. The obesogenic environment and widespread dietary and lifestyle changes are driven by urbanisation and rising income. These changes also affect children and may contribute to overnutrition^(3–5)^. Indonesia currently has one of the highest and fastest growing rates of childhood overweight and obesity in Southeast Asia (for review of the available evidence see ref. 6) with sex-specific risks^(7)^. The latest estimates from the 2018 National Basic Health survey indicate that nationwide 20 % of children of 5–12 years old are overweight or obese^(8)^. The prevalence of childhood overweight is higher in urban areas, reaching 29·2 % in Jakarta^(8)^.

Previous research has documented a variety of factors contributing to the development of childhood overweight and obesity, such as family socio-economic background, children’s energy intake and expenditure, as well as environmental, socio-cultural, and media influences. The apparent heterogeneity of findings from high-, middle- and low-income countries indicates the complex relationships between these factors, which may vary both between and within countries and households^(9,10)^. Family socio-economic markers (household income and parental education) could be both positively and negatively associated with childhood overweight and obesity^(11)^. While the growing consumption of unhealthy foods and corresponding overweight might be attributed to rising incomes^(12)^ (for Indonesia see refs. 5 and 13), the income–obesity link might change over time with a country’s economic development^(14)^ and advancement of nutrition transition^(11)^. Similarly, significant nutritional returns to maternal education were documented in LMIC^(15)^, while at the same time, the increase in female education and labour force participation might lead to an increasing prevalence of overweight and obesity^(12)^ (for Indonesia see refs. 5 and 16).

Emerging literature explores the potential co-occurrence of various factors associated with childhood overweight. However, to date, childhood obesity research has primarily focused on the joint influence of dietary and activity patterns^(17,18)^. The concurrent influence of physical and social environments on children’s healthy behaviours and weight status requires further consideration^(19)^. The available conceptualisations of this environment illustrate the interaction of numerous contextual factors operating at the macro (community) and micro (family) levels^(20)^. Previous food environment research in LMIC was predominantly concerned with the macro-level environment and dealt mostly with the availability of unhealthy foods and beverages in neighbourhoods and schools^(19)^. Family or parental influence was often used as a component of school-based programmes rather than a central target of the research or policy^(20)^. The home food environment is composed of a broad range of components, spanning from the availability and accessibility of healthy and unhealthy foods to parental attitudes and practices^(21,22)^. The complexity of factors, their interaction and their influence on children’s behaviours and health outcomes have been conceptualised in various models^(23,24)^. However, most food environment research has been conducted in high-income countries, and there is little evidence from LMIC, where the food environment and diets are under transition, and where the rising prevalence of childhood overweight requires urgent attention^(19)^. A summary of a scoping review of the available studies in LMIC is provided in online Supplementary Appendix A.

The present study contributes to childhood obesity research in LMIC by providing novel evidence of a comprehensive set of demographic, behavioural and environmental risk factors and their associations with children’s weight across its entire distribution using recent data on a focused high-risk population in the urban setting of an upper middle-income country.

## Conceptual framework

The ecological systems theory states that children’s behaviour occurs in and is influenced by ‘its ecological context; that is, in the actual environments in which human beings live their lives’^(25)^ (as put forward by Bronfenbrenner, p.794). This theory has been applied in public health interventions to assess the intersecting influences of cultural, social, economic, psychological and environmental factors on children’s health behaviours^(20)^.

The present study adopted the family ecological model that extends the ecological systems theory and focuses on the family as a domain for intervention^(20)^. The study retained some community (neighbourhood walkability and crime levels), policy (food labelling, school physical education and food policies), media (advertising to children) and organisational characteristics (school environment, job characteristics and work demand). The home environment is conceptualised as a broad range of social and physical factors within the family, such as food availability and routines, parental knowledge, and practices that promote or impede children’s health behaviours related to eating and physical activity and may contribute to an increased risk of overweight and obesity (for review of previous research see ref. 21).

**Fig. 1:**
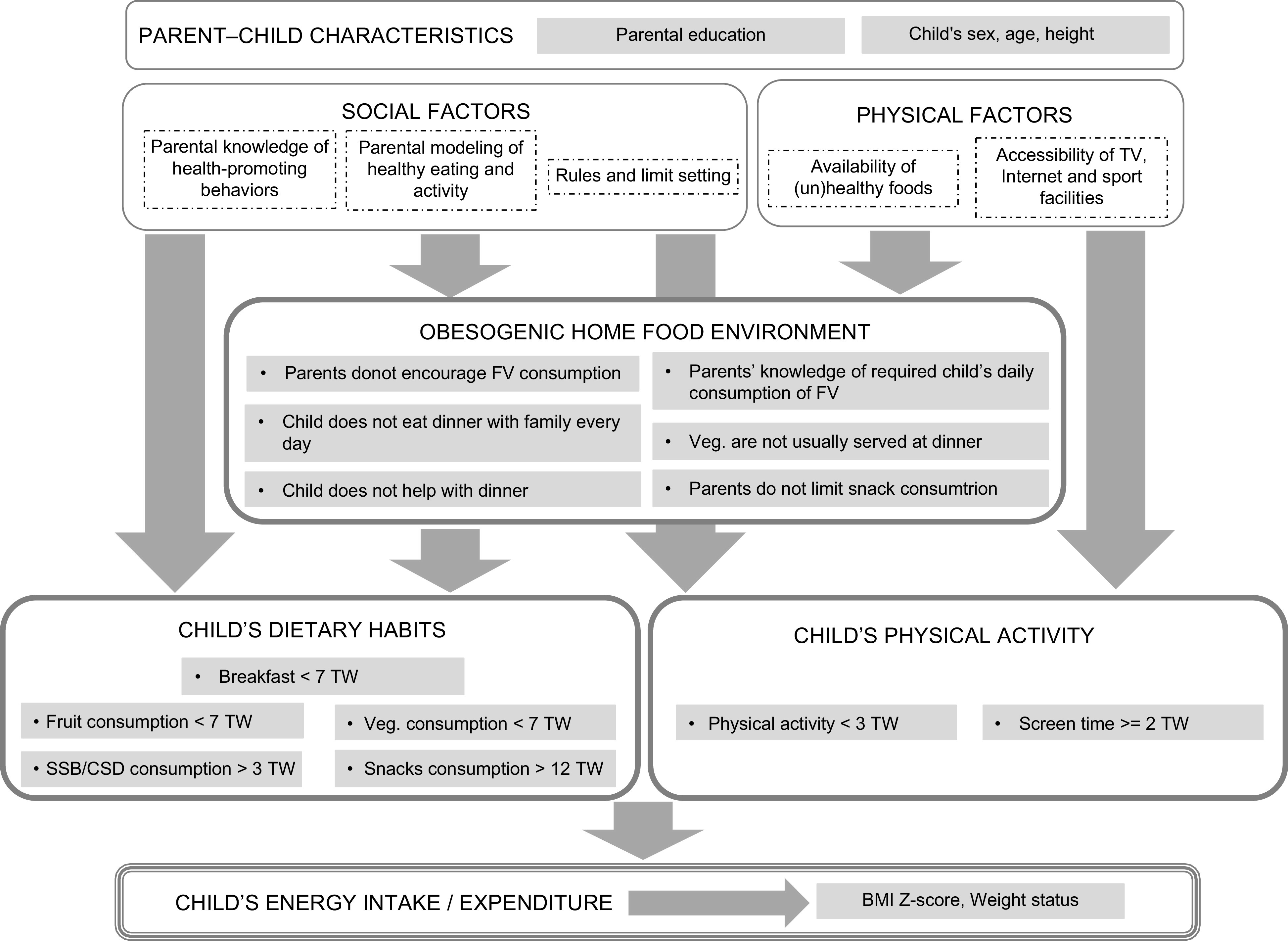
Conceptual framework. FV, fruit and vegetables; Veg., vegetables; TW, times a week; SSB/CSD, sugar-sweetened beverages/carbonated soft drinks

The conceptual framework (Fig. 1) includes three domains of behavioural (*dietary habits* and *physical activity*) and environmental (*obesogenic home food environment*) risk factors, each of which has a set of measurable dimensions. The domain of *dietary habits* includes measurements of children’s dietary intake, such as consumption of breakfast, fruits, vegetables, snacks, and sugar-sweetened and soft drinks. The *physical activity* domain comprises measurements of a child’s physical activity level and sedentary behaviour (screen time). The *obesogenic home food environment* is related to parental nutrition knowledge and family eating practices. All domains are associated with family social (parental influences) and physical (home environment) factors, as well as with parent–child socio-economic correlations. This conceptual framework informs the empirical specifications of this study.

## Methods

### Data collection and participants

The sample consisted of 1674 children aged 6–13 years in grades 2–5 from eighteen public primary schools in Central Jakarta, Indonesia. Schools were randomly sampled from a list of 279 schools in Central Jakarta in the Ministry of Education and Culture’s database (*rand* function in Excel), as described elsewhere^(26)^. School principals invited class teachers for a briefing session. Among the teachers present during the session, at least one class in each grade was selected by the principal and teachers to be included in the study. Children who were reported to have no history of food allergies and were available at school on a certain day participated in a separate snack choice experiment^(26)^. All but four parents/guardians of the participating children completed a self-administered parental survey (the English version of the survey questionnaire is provided in online Supplementary Appendix B) that was sent home along with an informed consent letter to obtain parental permission for the children to participate in the experiment. During the experiment, the school food environment was not formally assessed. However, as observed during the data collection, informal food vendors often operate near or even inside the school premises, offering an assortment of prepared foods and snacks to children.

The self-administered parental survey collected information on family socio-economic background, parental recall of children’s last 7 d consumption of various foods, physical activity, sedentary behaviour, home food environment, and parental practices. The survey instruments were obtained from existing validated tools such as the FFQ^(27,28)^ and Child Feeding Questionnaire^(29,30)^. A bilingual researcher translated the survey instruments into Bahasa Indonesia. Local experts then reviewed the translated instruments to ensure that the scales and selected food items were appropriate for the study settings. The survey instruments were pretested among thirteen randomly selected mothers of primary schoolchildren. Anthropometrics (height and weight) of students whose parents provided consent were measured by a trained nutritionist.

### Measures

#### Child’s height and weight

Two measurements were obtained for each child’s height and weight. Height and weight variables used in the analysis were constructed using average of these two measurements. Anthropometric data were missing for three children. BMI was calculated as the weight in kilograms divided by the squared height in metres. The respective BMI Z-score, adjusted for age and sex, was calculated using the WHO Child Growth Standards and WHO Reference 2007 data and the command *zanthro* ^(31)^ in STATA version 16.1 (StataCorp. LLC, College Station, TX, USA). The WHO Child Growth Standards (age- and sex-specific) cut-offs were applied to estimate the proportion of normal, overweight (BMI-for-age Z-score above +1 sd), and obese (BMI-for-age Z-score above +2 sd) children^(32,33)^. Children with extreme BMI values (equal to or greater than 5 sd from the mean for their age) were not excluded from the sample as they were accommodated by quantile regression analysis.

#### Socio-economic and demographic variables

The parental survey included information on the children’s age, sex, maternal and paternal income, and educational attainment. Parental income was estimated as the sum of the monthly salaries of the mothers and fathers. The variable was then dichotomised according to whether parental income was equal or lower than the minimum wage. The highest level of education attained by each parent was converted into years: 6 years for elementary school, 9 years for junior high school, 12 years for senior high school, 16 years for undergraduate studies and 18 years for master’s and PhD degrees.

#### Child’s dietary intake and physical activity

A score for the behavioural risk associated with an unhealthy diet was created for each child^(18)^. Four questions of the self-administered parental survey recorded the parental recall of the number of times a week the child consumed fruits (five items: orange, watermelon, papaya, mango and banana), vegetables (five items: carrot, spinach, kale, tomato and beans), snacks (five items: chips, sweet and savoury snacks, confectionery, biscuits, and ice cream) and sugar-sweetened beverages (four items: carbonated soft drinks, flavoured drinks, juice and sweetened tea). Additional fruits (apples, grapes, dragon fruit, snake fruit, durian, guava, etc.), vegetables (mustard green, bean sprout, beans, corn, broccoli, cabbage, etc.) and snacks (bread, fried food, pizza, pudding, cake, instant noodles, etc.) were reported by parents in the open question (*Other*) and used in the respective variables. No other items of sugar-sweetened beverages reported in the open question (*Other*) were different from the provided answer options. The place of consumption (home, school or elsewhere) was not specifically asked in the questions on food and beverages intake. One question captured the frequency of the child’s breakfast consumption over the last 7 d. The average weekly intake of the four food groups and breakfast consumption were used to compute a dietary risk score. The cut-off points^(34)^ were applied to create dichotomised variables indicating whether the child consumed less than one fruit (< 7 times a week) and one vegetable (< 7 times a week) per d, frequently consumed snacks (> 12 times a week) and sugar-sweetened beverages (> 3 times a week) and did not eat breakfast every day (< 7 times a week). The scores of the five components were summed to obtain the child’s overall dietary risk score.

The physical activity risk score was estimated using the number of times the child was running, playing a ball or cycling during the last 7 d, and the average number of hours per d that the child spent playing computer games, using the Internet, or watching TV. Additional physical activities (such as walking, playing badminton, volleyball, basketball, dancing, swimming and performing martial arts) were reported by parents in the open question (*Other*) and were also considered. Similar to the dietary risk score, the cut-off points for limited physical activity (< 3 times a week) and prolonged screen time (≥ 2 h per d) were applied^(34)^. The two scores were then combined to obtain the child’s overall physical activity risk score.

#### Home food environment

The obesogenic home food environment was measured using six components of parental self-reports on the encouragement of children’s fruit and vegetable consumption (one item; five-point Likert scale), frequency of children helping to prepare dinner (one item; number of times a week), eating dinner with the family (one item; number of times a week), having vegetables at dinner (one item; five-point Likert scale), parental knowledge of the guidelines for children’s fruit and vegetable intake (two items, number of servings per d), and restrictions on snack consumption (one item; five-point Likert scale). The choice of variables was informed by the conceptual framework and previous studies^(35–37)^. Six dichotomised and reverse-coded variables for each component of the home food environment were constructed to indicate whether the parents failed to encourage the child’s fruit and vegetable consumption, the child did not help prepare dinner (< 1 time a week), the child did not eat dinner with the family every day (< 7 times a week), vegetables were not usually served at dinner, parental knowledge of the required child’s fruit and vegetable intake did not meet the guidelines of at least five servings of fruits and vegetables a day, as recommended by the WHO^(38)^ and Indonesian Ministry of Health^(39)^, and parents did not restrict the child’s snack consumption. The scores of the six components were summed to obtain the overall obesogenic home food environment score.

### Statistical analysis

Means and standard deviations were used to describe continuous variables, and frequencies and percentages were used for categorical variables. Fisher’s exact test was used to determine if there was a significant association between categorical variables, and *t* test was performed for continuous variables. Missing observations of children’s age (*n* 131) and maternal education (*n* 63) were interpolated using the class and pooled sample means, respectively. The indicator of parental income was not used in the analysis for two reasons. First, 14·0 % of observations were missing due to the potential sensitivity of reporting, as was found in the pretest. Second, the variable was strongly and negatively correlated with years of maternal education. Parental education was found to be associated with the higher socio-economic status of households in many LMIC^(40)^. As in Indonesia mothers are the main caregivers^(41)^, the indicator of maternal years of education was used in the empirical specification. Missing observations for behavioural and environmental risk factors were excluded from the analysis (listwise deletion). Spearman’s rank correlation was used to assess the strength of relationship between the risk scores and their components.

The first set of analyses tested the associations between overweight/obesity status and children’s demographics and exposure to behavioural and environmental risks described in the conceptual framework. The relationships between child–parent characteristics and three risk scores (diet, physical activity and home food environment) that captured various risk factors were modelled using logistic regression. As the influence of the risk factors may vary depending on the child’s weight status^(6)^, the second set of analyses examined the associations between behavioural and environmental risk scores and children’s weight across five percentiles (10th, 25th, median, 75th and 95th percentiles) of the standardised BMI-for-age Z-score distribution using a quantile regression model^(42)^ (*sqreg* command in STATA). Standard errors stemmed from 2000 bootstrap replications. The model included controls for children’s age, height and maternal education and was fitted for the pooled sample as well as separately for boys and girls. The additional analysis explored the associations between the components that might contribute to the obesogenic home food environment and children’s consumption of fruits, vegetables, and snacks. In all analyses, the statistical significance of the findings was highlighted using a 5 % significance cut-off.

## Results

### Sample characteristics

The socio-economic characteristics of the children and their parents are shown in Table 1. The mean age was 9·5 years. The average height was 134·2 cm, and the average weight was 32·3 kg. Almost half (48·0 %) of the 1674 children were male. Most parents (77·0 %) had a combined income equal to or lower than the minimum wage of Rp3,941 000 (US$284). The average education of parents was about 11·2 years and did not differ across parents’ or children’s sexes.

**Table 1:** Descriptive statistics of children in selected primary schools in Jakarta

Variable	Total sample					By sex		
	Obs	Mean	Sth. dev.	Min	Max	Male	Female	Test for differences in means, *P* values
Child is male, d	1674	0·48		0·0	1·0			
Child’s age, years	1674	9·51	1·16	6·0	13·0	9·50	9·51	0·817
Child’s weight	1671	32·31	10·34	15·10	76·20	32·16	32·44	0·578
Child’s height	1671	134·18	9·48	106·50	161·0	133·51	134·79	0·005
Child’s BMI Z-score (WHO, no cut-off)	1671	0·19	1·61	−5·85	4·16	0·23	0·16	0·411
Child is overweight or obese, d	1671	0·31		0·0	1·0	0·34	0·29	0·039
Child is obese, d	1671	0·17		0·0	1·0	0·21	0·12	0·000
Parental income is equal or lower than min. wage, d	1446	0·77		0·0	1·0	0·78	0·77	0·706
Maternal education in years	1674	10·97	2·69	6·0	18·0	10·87	11·05	0·169
Paternal education in years	1674	11·38	2·38	6·0	18·0	11·30	11·44	0·231
Child’s FV consumption – times a week L7D	1565	12·29	9·34	0·0	77·0	11·55	12·97	0·003
Child’s fruit consumption – times a week L7D	1543	6·99	6·00	0·0	53·0	6·60	7·33	0·017
Child’s vegetables consumption – times a week L7D	1537	5·51	4·82	0·0	42·0	5·17	5·80	0·010
Child’s FV daily consumption meets the guidelines (>= 5 a day), d	1565	0·03		0·0	1·0	0·03	0·03	0·375
Parental knowledge of the required child’s daily FV consumption	1452	3·57	1·98	0·0	22·0	3·60	3·54	0·569
Parents encourage child’s FV consumption, d	1510	0·84		0·0	1·0	0·84	0·85	0·619
Child’s snacks consumption – times a week L7D	1563	9·80	6·59	0·0	40·0	9·43	10·12	0·040
Parents limit child’s snacks consumption, d	1524	0·84		0·0	1·0	0·84	0·84	0·889
Child’s sweetened drinks consumption – times a week L7D	1551	5·54	4·05	0·0	28·0	5·23	5·82	0·004
Child eats breakfast – times a week L7D	1430	4·44	2·73	0·0	7·0	4·57	4·33	0·099
Child helps prepare dinner – times a week L7D	1430	1·60	2·33	0·0	7·0	1·33	1·83	0·000
Child eats dinner with family – times a week L7D	1430	4·63	2·79	0·0	7·0	4·54	4·71	0·251
Vegetables are usually served at dinner, d	1508	0·33		0·0	1·0	0·32	0·34	0·661
Child’s physical activity – times a week L7D	1513	4·08	3·68	0·0	22·0	5·27	2·99	0·000
Child’s daily screen time – hours a day L7D	1505	5·09	2·95	0·0	18·0	5·38	4·83	0·000

d, dummy variable; L7D, last 7 d; FV, fruit and vegetables.

Differences of the means assessed by using the Fisher’s exact test for categorical variables and *t* test for continuous variables.

### Childhood overweight and obesity

Approximately one-third (31·0 %) of the children in the sample were overweight or obese, whereas 17·0 % were obese (Table 1). Obesity was higher in boys (21·0 %) than in girls (12·0 %) (two-tailed *P* < 0·0001). The average BMI-for-age Z-score was 0·19 sd with a minimum of −5·85 and a maximum of 4·16.

### Children’s dietary intake and activity

On average, the children consumed at least one serving of fruit and one serving of vegetables daily (7·0 and 5·5 times a week, respectively, Table 1). Boys ate fruits and vegetables significantly less frequently than girls did (both *P* < 0·05). Children consumed snacks and sugar-sweetened beverages almost every day (9·8 and 5·5 times a week, respectively), and the consumption frequency was higher among girls (both *P* < 0·05). A majority of parents (84·0 %) agreed that they limit snack consumption (*‘I have to be sure that my child does not eat too many sweet/savoury snacks–chips, chocolate, candy, ice cream*). Additionally, most parents (93 %, not reported in the table) were aware of children’s food intake at school as children indicated in their responses to the question *‘Do your parents often ask what food you eat at school?’* (the English version of the children’s questionnaire is provided in online Supplementary Appendix B). On average, children ate breakfast and participated in family dinners approximately four times a week. Children rarely helped prepare dinner (on average 1·6 times a week, girls more frequently; *P* < 0·0001).

Children in the sample spent 5·1 h a day watching TV, playing games on a computer/mobile, or using the Internet. Boys had a significantly longer screen time than girls (*P* < 0·0001). Children went outside to run, bike or play ball on average 4·1 times a week, and boys were significantly more physically active than girls (*P* < 0·0001).

### Behavioural and environmental risk factors

Table 2 presents the indicators for behavioural (i.e. diet and physical activity) and environmental risk scores as well as the prevalence of their components.

**Table 2:** Prevalence and scores of behavioural and environmental risk factors

Scores/indicators	Score, *pts/*prevalence, %				
	Pooled sample	95 % CI	Boys	Girls	Test for differences in means, *P* values
Dietary risk score (scale 0–5):	2·57		2·53	2·60	0·450
Fruit consumption < 7 times a week, d	57·49	55·02, 59·96	61·04	54·30	0·009
Vegetables consumption < 7 times a week, d	71·70	69·44, 73·95	74·31	69·35	0·031
Drinks consumption > 3 times a week, d	61·77	59·35, 64·19	58·78	64·46	0·024
Snacks consumption > 12 times a week, d	29·69	27·42, 31·95	27·02	32·08	0·031
Breakfast < 7 times a week, d	54·69	52·10, 57·27	52·62	56·49	0·151
No risk	2·87		3·13	2·63	
4–5 risk factors	18·82		17·52	20·00	
Physical activity risk score (scale 0–2):	1·36		1·24	1·46	0·000
Physical activity < 3 times a week, d	44·81	42·30, 47·32	32·19	56·49	0·000
Screen time >= 2 h a day, d	94·55	93·40, 95·70	96·47	92·85	0·002
No risk	3·05		3·38	2·74	
Two factors	35·96		25·66	45·37	
Obesogenic home food environment score (scale 0–6):	2·51		2·57	2·46	0·201
Parents do not encourage FV consumption, d	15·56	13·73, 17·39	16·06	15·11	0·619
Child does not help with dinner, d	53·85	51·26, 56·43	61·17	47·44	0·000
Child does not eat dinner with family every day, d	48·53	45·94, 51·13	50·23	47·05	0·243
Vegetables are not usually served at dinner, d	66·98	64·60, 69·35	67·60	66·42	0·661
Parental knowledge of the required child’s daily FV consumption < 5, d	78·93	76·83, 81·03	77·84	79·87	0·367
Parents do not limit snacks consumption, d	16·14	14·29, 17·99	16·34	15·96	0·889
No risk	3·94		4·38	3·54	
2–3 factors	54·96		53·94	55·89	
5–6 factors	5·50		6·01	5·03	

d, dummy variable; FV, fruit and vegetables.

Differences of the means assessed by using the Fisher’s exact test for categorical variables.

The average dietary risk score in the pooled sample was 2·6 on a scale of 0 to 5. There was no sex difference in the overall score (two-tailed *P* = 0·450), but there were in all its components excluding breakfast consumption. Most children (71·7 %) did not consume vegetables daily, and slightly more than half did not eat breakfast and fruits every day (54·7 % and 57·5 %, respectively). Excessive consumption of snacks was reported in one-third of children (29·7 %). Two-thirds of the children frequently (more than three times a week) consumed sugar-sweetened beverages (61·8 %). Only 2·9 % of the children scored 0 points, while 18·8 % had four or five dietary risk factors.

The average risk score of physical activity was 1·4 on a scale from 0 to 2 but was more prominent among girls (two-tailed *P* < 0·0001). Of the children, 94·6 % had two or more hours of screen time daily, and 44·8 % did not participate in active sports or games at least three times a week. The combination of a low level of physical activity and high level of sedentary behaviour (a maximum score of 2) was achieved by 36·0 % of the children and almost half of the girls (45·4 %).

The score for obesogenic home food environment was 2·5 out of 6, without significant differences across sex (two-tailed *P* = 0·201). Only 3·0 % of the sample met the WHO^(38)^ and Indonesian Ministry of Health^(39)^ guidelines for the five servings of fruits and vegetables daily. An average parent in the sample believed that the child should consume 3·6 servings of fruits and vegetables a day. Most parents (84·0 %) reported that they encouraged their children to consume fruits and vegetables, and 33·0 % agreed with the statement that vegetables are usually served at dinner. At the same time, every sixth parent did not encourage their children to eat fruits and vegetables (15·6 %), and most parents were unaware of the required child’s daily consumption of five servings of fruits and vegetables (78·9 %). Moreover, 67·0 % of parents did not usually serve vegetables at dinner, and 16·1 % reported that they did not limit their children’s snack consumption. A large proportion of the children did not regularly help prepare or eat dinner with their families (53·9 % and 48·5 %, respectively). Only 3·9 % of the children were not exposed to any environmental risk factors, while 5·5 % of the children scored a maximum of 5–6 points. Approximately two-thirds of the children (55·0 %) faced two or three negative environmental components.

Each risk score was highly correlated with the factors underlying other risk scores (Table 3). The dietary risk score (five items, *α* = 0·54) was strongly associated with the obesogenic home food environment and its components, such as parental encouragement of fruit and vegetable consumption, parental limits of snack consumption, serving vegetables at dinner, and having regular family dinners. The physical activity risk score (two items, *α* = 0·15) was correlated with children’s consumption of fruits, vegetables, drinks and snacks. The obesogenic home food environment score (six items, *α* = 0·31) was strongly associated with irregular consumption of fruits, vegetables, sugar-sweetened beverages and breakfast as well as with the level of children’s physical activity. Low values of Cronbach’s alpha (not reported in the table) are suggestive of the heterogeneity in latent sources of risk within the same domain.

**Table 3:** Correlation of risk scores and their components

	Dietary risk score (scale 0–5)	Physical activity risk score (scale 0–2)	Obesogenic home food environment score (scale 0–6)
Dietary risk score (scale 0–5):			
Fruit consumption < 7 times a week, d		0·15***	0·19***
Vegetables consumption < 7 times a week, d		0·13***	0·23***
Drinks consumption > 3 times a week, d		−0·11***	−0·12***
Snacks consumption > 12 times a week, d		−0·09**	−0·05
Breakfast < 7 times a week, d		0·04	0·19***
Physical activity risk score (scale 0–2):	0·06*		
Physical activity < 3 times a week, d	0·07*		0·11***
Screen time >= 2 h a day, d	−0·02		−0·04
Obesogenic home food environment score (scale 0–6):	0·21***	0·09**	
Parents *do not* encourage FV consumption, d	0·14***	0·02	
Child *does not* help with dinner, d	0·04	0·06	
Child *does not* eat dinner with family every day, d	0·20***	0·08**	
Vegetables *are not* usually served at dinner, d	0·06*	0·00	
Parental knowledge of the required child’s daily FV consumption < 5, d	0·09**	0·06*	
Parents *do not* limit snacks consumption, d	0·08**	0·05	

d, dummy variable; FV, fruit and vegetables.

Number of paired ranks *n* 1121. Spearman’s rank correlation.

*P < 0·05,

**P < 0·01,

***P < 0·001.

### Risk factor relationships and their association with childhood overweight and obesity

Table 4 presents the estimates of the logit models that assess associations between the odds of childhood overweight or obesity and risk factors, including child’s (sex, age and height) and maternal (years of education) characteristics, and behavioural (child’s diet and physical activity) and environmental risk factors at home (the estimates of a linear probability model, showing qualitatively similar results, are provided in online Supplementary Table SC1 in Supplementary Appendix C). There were strong positive associations between the child’s male sex (aOR = 1·67; 95 % CI 1·30, 2·14) and height (aOR = 1·16; 95 % CI 1·14, 1·18 with weight status (Column 1). Every year of age reduced the odds of being overweight or obese by a factor of 0·43 (95 % CI 0·37, 0·50) (Column 1). Maternal years of education displayed a positive but insignificant association with childhood overweight in all models. The influence of behavioural risk factors (dietary and physical activity risk scores) was statistically insignificant (Columns 1, 2 and 3). The score for the obesogenic home food environment significantly increased the odds of childhood overweight or obesity (OR = 1·11; 95 % CI 1·01, 1·22) (Column 4). Similar results were obtained when estimating a model that included seventeen school dummy variables to control for the school environment (online Supplementary Table SC2 in Supplementary Appendix C).

**Table 4:** Risk factors of overweight and obesity among children in selected primary schools in Jakarta

	(1)		(2)		(3)		(4)	
	aOR	95 % CI	aOR	95 % CI	aOR	95 % CI	aOR	95 % CI
Child is male, *d*	1·67***	1·30, 2·14	1·67***	1·31, 2·11	1·66***	1·30, 2·13	1·61***	1·27, 2·04
Child’s age, years	0·43***	0·37, 0·50	0·41***	0·35, 0·48	0·43***	0·37, 0·50	0·41***	0·35, 0·48
Child’s height, cm	1·16***	1·14, 1·18	1·17***	1·14, 1·19	1·16***	1·14, 1·18	1·17***	1·14, 1·19
Mother’s years of education	1·03	0·98, 1·08	1·03	0·99, 1·08	1·02	0·98, 1·07	1·03	0·99, 1·08
Child’s dietary risk score (0–5)	1·04	0·93, 1·17	1·08	0·97, 1·21				
Child’s physical activity risk score (0–2)	1·03	0·82, 1·28			1·04	0·83, 1·29		
Obesogenic home food environm. score (0–6)	1·11*	1·00, 1·23					1·11*	1·01, 1·22
Number of observations	1538		1628		1547		1631	

d, dummy variable; environm., environment.

Logistic regression model. Robust standard errors. Dependent variable: a binary overweight indicator based on the BMI-for-age Z-score above +1 sd according to the WHO Child Growth Standards^(32,33)^. All specifications included a dummy for missing observations of age and maternal years of education, which were interpolated with the sample mean.

*P < 0·05,

**P < 0·01,

***P < 0·001.

Behavioural (dietary and physical activity) risk scores were not associated with children’s weight across five percentiles of the standardised BMI-for-age Z-score distribution in the pooled sample (Table 5). The obesogenic home food environment risk core was significantly associated with the children’s BMI-for-age Z-score at the 75th and 90th percentiles (obesity) (*P* = 0·022 and 0·023, respectively). Maternal education was positively and significantly associated with children’s BMI-for age Z-score at the median (*P* = 0·026). Male sex was predictive of higher BMI-for-age Z-scores (75th and 90th percentiles, both *P* < 0·0001). This finding underlines the rationale for estimating the model separately for girls and boys. Table 6 provides the detailed estimates of the quantile regression model by sex. The dietary and physical activity risk scores were not associated with the child’s BMI across its Z-score distribution (Panels A and B). The coefficients of the obesogenic home food environment at the 75th and 90th percentiles were somewhat larger for boys than for girls but were not precisely estimated (*P* = 0·105 and 0·069, respectively).

**Table 5:** Child’s BMI Z-score and behavioural and environmental risk scores

	OLS	10th percentile	25th percentile	Median	75th percentile	90th percentile
Child is male, d	0·205**	−0·272*	−0·235*	0·202	0·583***	0·721***
	0·075	0·132	0·093	0·104	0·129	0·109
Child’s age, years	−0·643***	−0·551***	−0·565***	−0·622***	−0·774***	−0·675***
	0·045	0·078	0·064	0·058	0·060	0·070
Child’s height, cm	0·111***	0·085***	0·100***	0·118***	0·129***	0·109***
	0·005	0·010	0·007	0·007	0·006	0·008
Mother’s years of education	0·010	−0·022	0·007	0·034*	0·006	−0·007
	0·014	0·020	0·020	0·015	0·020	0·024
Child’s dietary risk score (0–5)	−0·026	0·009	0·005	−0·037	−0·009	−0·002
	0·035	0·055	0·050	0·045	0·054	0·050
Child’s physical activity risk score (0–2)	−0·010	−0·174	0·007	0·046	0·012	0·068
	0·066	0·119	0·081	0·086	0·107	0·106
Obesogenic home food environm. score (0–6)	0·051	−0·053	0·004	0·030	0·109*	0·110*
	0·032	0·053	0·043	0·039	0·048	0·048
Constant	−8·799***	−6·923***	−8·868***	−10·222***	−9·318***	−6·691***
	0·547	1·039	0·645	0·714	0·683	0·824
R2	0·24	0·09	0·11	0·16	0·18	0·18

d, dummy variable; environm., environment.

*n* 1538. Dependent variable: BMI-for-age Z-score that was calculated using the WHO Child Growth Standards and WHO Reference 2007 data^(31)^. Column 1: pooled linear regression model. Robust standard errors. Columns 2–6: quantile regression model. Bootstrapped standard errors (2000 replication). Pseudo-R-squared values are reported for quantile regression estimates. Both models included a dummy for missing observations of age and maternal years of education, which were interpolated with the sample mean.

*P < 0·05,

**P < 0·01,

***P < 0·001.

**Table 6:** Child’s BMI Z-score and behavioural and environmental risk scores, by sex

Panel A: boys						
	OLS	10th percentile	25th percentile	Median	75th percentile	90th percentile
Child’s age, years	−0·606***	−0·542***	−0·475***	−0·584***	−0·755***	−0·625***
	0·064	0·114	0·107	0·075	0·090	0·087
Child’s height, cm	0·123***	0·098***	0·097***	0·138***	0·154***	0·102***
	0·008	0·019	0·012	0·010	0·009	0·012
Mother’s years of education	0·014	−0·035	0·003	0·032	0·010	−0·007
	0·024	0·035	0·027	0·031	0·033	0·035
Child’s dietary risk score (0–5)	−0·049	−0·118	0·025	−0·053	−0·036	−0·029
	0·059	0·105	0·073	0·076	0·084	0·073
Child’s physical activity risk score (0–2)	−0·048	−0·137	−0·096	−0·184	0·175	0·084
	0·113	0·218	0·143	0·135	0·172	0·161
Obesogenic home food environm. score (0–6)	0·055	0·014	0·036	0·029	0·112	0·134
	0·049	0·070	0·066	0·063	0·069	0·073
Constant	−10·579***	−8·803***	−9·450***	−12·709***	−12·593***	−5·522***
	0·907	1·945	1·093	1·077	1·248	1·522
R2	0·24	0·06	0·09	0·16	0·18	0·14

Panel B: girls						
	OLS	10th percentile	25th percentile	Median	75th percentile	90th percentile
Child’s age, years	−0·675***	−0·572***	−0·610***	−0·660***	−0·841***	−0·828***
	0·064	0·096	0·080	0·072	0·082	0·091
Child’s height, cm	0·102***	0·078***	0·093***	0·109***	0·118***	0·124***
	0·007	0·012	0·009	0·008	0·008	0·010
Mother’s years of education	0·008	−0·031	0·007	0·022	−0·001	0·026
	0·016	0·024	0·027	0·018	0·026	0·032
Child’s dietary risk score (0–5)	−0·001	0·063	0·008	−0·014	0·053	0·024
	0·042	0·068	0·073	0·054	0·061	0·066
Child’s physical activity risk score (0–2)	0·019	−0·190	0·091	0·044	−0·079	0·032
	0·077	0·138	0·108	0·099	0·120	0·149
Obesogenic home food environm. score (0–6)	0·043	−0·065	−0·021	0·033	0·050	0·096
	0·040	0·081	0·053	0·045	0·059	0·065
Constant	−7·396***	−5·764***	−7·525***	−8·646***	−7·117***	−7·666***
	0·663	1·181	0·936	0·883	0·709	1·048
R2	0·26	0·11	0·13	0·17	0·19	0·18

d, dummy variable; environm., environment.

*n* 732 (boys) and 806 (girls). Dependent variable: BMI-for-age Z-score that was calculated using the WHO Child Growth Standards and WHO Reference 2007 data^(31)^. Column 1: linear regression model. Robust standard errors. Columns 2–6: quantile regression model. Bootstrapped standard errors (2000 replication). Pseudo-R-squared values are reported for quantile regression estimates. Both models include a dummy for missing observations of age and maternal years of education, which were interpolated with the sample mean.

*P < 0·05,

**P < 0·01,

***P < 0·001.

Additional analysis of the associations between the components of the obesogenic home food environment and children’s fruit, vegetable, and snack consumption is provided in Table 7. Children whose parents did not encourage fruit and vegetable consumption had lower odds of eating at least one serving of them daily (aOR = 0·40; 95 % CI 0·27, 0·58). Similarly, child’s involvement in dinner preparation (aOR = 0·51; 95 % CI 0·38, 0·68) and inadequate parental knowledge of the required fruit and vegetable intake (aOR = 0·67; 95 % CI 0·46, 0·96) reduced the odds of everyday consumption of fruit and/or vegetable. Higher maternal education (aOR = 0·86; 95 % CI 0·74, 0·98), lack of parental encouragement (aOR = 0·11; 95 % CI 0·01, 0·89), irregular family dinners (aOR = 0·31; 95 % CI 0·14, 0·68) and unavailability of vegetables at dinner (aOR = 0·35; 95 % CI 0·18, 0·69) decreased the odds of meeting the recommended fruit and vegetable intake of five or more servings per d^(38,39)^. At the same time, children whose parents did not limit their children’s snack consumption were 3·5 times more likely to consume five or more fruits and vegetables daily (aOR = 3·47; 95 % CI 1·53, 7·87). Interestingly, children who did not help prepare dinner were less likely to snack every day (aOR = 0·64; 95 % CI 0·49, 0·82) and 1·4 times more likely to meet the guidelines of restricted (less than 12 snacks per week) consumption of snacks (aOR = 1·37; 95 % CI 1·06, 1·76). Moreover, as Table 8 illustrates, parental and children’s responses to similar questions were strongly correlated (the English version of the children’s questionnaire is provided in online Supplementary Appendix B).

**Table 7:** Child’s fruit, vegetables, and snacks consumption and the components of obesogenic home food environment

	Fruit and vegetable consumption				Snack consumption			
	At least 1 FV daily (mean = 0·70)		Meets guidelines (>= 5 FV a day) (mean = 0·03)		At least 1 snack daily (mean = 0·62)		Meets guidelines (<= 12 snacks a week) (mean = 0·70)	
	OR	95 % CI	OR	95 % CI	OR	95 % CI	OR	95 % CI
Child is male, d	0·76	0·58, 1·00	0·73	0·38, 1·43	0·94	0·73, 1·20	1·23	0·96, 1·59
Child’s age, years	0·86	0·73, 1·01	1·25	0·92, 1·70	0·99	0·86, 1·15	0·97	0·85, 1·11
Child’s height, cm	1·01	0·99, 1·03	0·94**	0·90, 0·98	0·98	0·97, 1·00	1·03**	1·01, 1·04
Mother’s years of education	0·98	0·93, 1·04	0·86*	0·74, 0·98	0·98	0·94, 1·03	1·02	0·97, 1·07
Parents *do not* encourage FV consumption, d	0·40***	0·27, 0·58	0·11*	0·01, 0·89	0·74	0·52, 1·05	1·45	1·00, 2·12
Child *does not* help with dinner, d	0·51***	0·38, 0·68	0·68	0·33, 1·37	0·64***	0·49, 0·82	1·37*	1·06, 1·76
Child *does not* eat dinner with family every day, d	0·81	0·61, 1·06	0·31**	0·14, 0·68	0·96	0·75, 1·23	1·28	1·00, 1·65
Vegetables *are not* usually served at dinner, d	1·05	0·77, 1·42	0·35**	0·18, 0·69	1·22	0·93, 1·60	0·83	0·63, 1·08
Parental knowledge of the required child’s daily FV consumption < 5, d	0·67*	0·46, 0·96	0·53	0·27, 1·04	0·95	0·70, 1·28	1·10	0·82, 1·48
Parents *do not* limit child’s snacks consumption, d	1·45	0·96, 2·22	3·47**	1·53, 7·87	0·98	0·69, 1·39	0·72	0·51, 1·02

d, dummy variable; FV, fruit and vegetables.

Logistic regression models. Robust standard errors. *n* 1188. Dependent variables: Column 1 – child consumes at least one serving of fruit or vegetable a day; Column 2 – a binary indicator of child’s daily fruit and vegetable intake that meets the recommended five or more servings; Column 3 – child consumes at least one snack a day; Column 4 – a binary indicator of a child’s weekly consumption of snacks that meets the restriction of less than 12. All specifications included a dummy for missing observations of age and maternal years of education, which were interpolated with the sample mean.

*P < 0·05,

**P < 0·01,

***P < 0·001.

**Table 8:** Correlation of parental and children’s responses

Variable	Parental survey					Children’s quiz					rs
	Obs	Mean	Std. dev.	Min	Max	Obs	Mean	Std. dev.	Min	Max	
Child’s FV consumption daily	1565	1·76	1·33	0·0	11·0	1670	4·86	1·93	1·0	10·0	0·11***
Child’s fruit consumption daily	1543	1·00	0·86	0·0	7·57	1668	2·87	1·30	0·0	5·0	0·11***
Child’s vegetables consumption daily	1537	0·79	0·69	0·0	6·0	1652	2·01	1·08	1·0	5·0	0·07*
Parents *do not* encourage FV consumption, d	1510	0·16		0·0	1·0	1662	0·12		0·0	1·0	0·06*
Vegetables *are not* usually served at dinner, d	1508	0·67		0·0	1·0	1671	0·54		0·0	1·0	0·03

d, dummy variable; FV, fruit and vegetables, rs, Spearman’s rank correlation coefficient.

Number of paired ranks *n* 1398. Spearman’s rank correlation.

*P < 0·05,

**P < 0·01,

***P < 0·001.

## Discussion

The present study examined the associations of behavioural and environmental risk factors with childhood overweight and obesity among 6–13 years old children from public primary schools in Central Jakarta, Indonesia. The findings emphasise childhood overweight and obesity in Indonesia as an emerging and important public health issue that requires policy attention. Of the children, 31·0 % were overweight and more boys (21·0 %) than girls (12·0 %) were obese. Overall, childhood overweight and obesity in the present study were higher than the 15·6 % prevalence among 6–12-year-old children reported in a recent study by Oddo and colleagues^(4)^ and a 16·0 % prevalence in a nationally representative sample of young adolescents in Agustina et al.^(7)^. The higher prevalence of obesity among boys than that among girls conforms with the findings of previous research in Indonesia^(5,7)^.

Sex heterogeneity was also found in the children’s dietary habits and physical activity levels. Overall, only a few children met the recommendations for daily fruit and vegetable intake, and the average fruit and vegetable consumption by boys was significantly lower than that of girls. Girls consumed more snacks and sugar-sweetened drinks than boys. More than half of the children did not eat breakfast daily. Although boys spent more time running, cycling or playing a ball, they also spent significantly longer hours watching TV, using the Internet or playing computer games. In general, almost all children had two or more hours of daily screen time. In line with previous studies in Indonesia, girls were less physically active than boys^(7,43,44)^ and had a comparatively similar level of screen time^(45)^. Previous study in Indonesia explained the lower level of girls’ physical activity by the cultural restrictions of girls’ participation in sports (‘girls should not be sporty’)^(44)^ as well as by the domestic activities they have to undertake^(7)^. Children’s low levels of physical activity and prolonged screen time were correlated with low intake of fruits and vegetables. This finding corresponds with previous research in high-income settings that reported a link between children’s sedentary behaviour and unhealthy eating^(37)^.

The use of behavioural and environmental risk scores that account for the co-occurrence of various risk factors of childhood overweight is one of the innovations of the present study. To the best of the authors’ knowledge, to date, only one study in LMIC has explored the prevalence of dietary and inactivity risk scores from the sets of related factors^(43)^. Although it was not possible to compare the findings directly owing to the use of different factors and cut-off points, the results of the present study resemble those of previous reports. The average dietary risk score was 2·6 (scale 0–5) and sex homogeneous. Previous research reported a 79·7 % prevalence of unhealthy food intake among boys and 78·2 % among girls^(43)^. Similarly, the average risk score for physical activity was 1·4 (scale 0–2) and somewhat higher for girls as compared with 84·5 %/64·5 % and 86·1 %/64·7 % prevalence of physical activity/sedentary behaviour among girls and boys reported by Sultana and colleagues^(43)^.

Research in high-income countries has emphasised the importance of the home food environment for children’s healthy eating^(20)^, especially among younger children^(36)^. The present study found that the overall obesogenic home food environment score was correlated with a child’s irregular consumption of fruits, vegetables and breakfast, as well as with a low level of physical activity. Although most parents in the present study reported encouragement of their children’s fruit and vegetable intake and restriction of snack consumption, many were not familiar with dietary guidelines and did not provide vegetables at dinner. About half of the children did not help prepare and had dinner with their family on a regular basis.

Childhood overweight in the present study was strongly associated with children’s (male) sex, taller stature and younger age. A study of Indonesian adolescents from the 2013 wave of the Basic Health Survey (*n* 155 645) reported a higher overweight prevalence among 10–14-year-olds as compared with 15–18-year-old children^(45)^. That trend persisted in 2018 as reported in a recently published study by Agustina and colleagues^(7)^. Rachmi et al. analysed the data from four cross-sectional waves of the Indonesian Family Life Survey (*n* 4101) and found that younger children (2·0–2·9-year-old) had a higher prevalence of overweight^(16)^. While cultural norms that favour boys over girls^(5)^ may translate into parental feeding practices and consequent overnutrition of boys, sex- and age-specific concerns about body image^(7)^ may also play a role in higher prevalence of overweight and obesity among boys and younger children in Indonesia.

The risk of being overweight increased with higher maternal education after adjusting for other predictors of obesity such as diet and home food environment. To date, there has been mixed evidence on the influence of maternal education on children’s weight status. Previous research in developed countries has often used maternal education as a proxy for overall knowledge about healthy diet and other health-promoting behaviours^(15)^. This expectation was not supported by evidence from developing countries, where previous studies found a positive association between parental education and childhood overweight and obesity^(18,46)^. Educated mothers may have a higher socio-economic status and work-time pressure and may tend to replace nutritious home-cooked meals with ready-to-eat processed foods and snacks^(5,13,40)^. Conversely, a recent study of malnutrition risk factors in two Indonesian districts (*n* 2160) reported a negative association between maternal education and adolescent overweight^(47)^. Further research is required to explore the associations between parental characteristics and childhood overweight and obesity in Indonesia.

In line with previous research in ASEAN countries^(48)^ and Indonesia^(47)^, the dietary and physical activity risk scores in this study were not associated with the children’s weight status. Exposure to an obesogenic home food environment resulted in higher odds of being overweight. However, the studied risk factors differed across the five quantiles of the standardised BMI-for-age Z-score distribution. Maternal education was positively and significantly associated with children’s BMI-for-age Z-scores at the median. The child’s male sex was predictive of a higher BMI-for-age Z-score (75th and 90th percentiles). Exposure to the obesogenic food environment at home was strongly and significantly associated with a high BMI (75th and 90th percentiles of the Z-score distribution) in the pooled sample.

An adequate level of consumption of fruits and vegetables, together with a limited intake of nutrient-poor processed foods, should be the main component of a healthy diet^(49)^. Parental knowledge, encouragement and family routines play an important role in promoting children’s healthy behaviours^(50)^. The findings of this study were consistent with this notion. Home food environment was found to correlate with the child’s diet, particularly the intake of fruits, vegetables and breakfast. Children whose parents did not encourage fruit and vegetable intake consumed fewer fruits and vegetables. Children who lacked parental encouragement were not served vegetables at dinner and did not regularly eat dinner with their family were less likely to meet the recommended five servings of fruit and vegetables per d. At the same time, children whose parents did not limit snack consumption had a much greater chance of consuming five or more fruits and vegetables a day than children with restrictive parents. Previous research in Southeast Asia reported positive associations between parental control, restriction of food consumption and childhood overweight^(6)^. Parental practices are shaped by socio-cultural norms and beliefs especially in contexts where awareness of the negative influence of environmental factors and detrimental consequences of childhood overweight is low^(41)^.

This study had some limitations. First, it used self-reported recall data. Parental responses might suffer from social desirability bias by over-reporting healthy behaviours and under-reporting unhealthy behaviours. Despite that the survey instruments were obtained from existing validated tools^(27–30)^, the number of dietary and activity components was limited due to time and logistical constraints. The respondents’ self-reports to frequency questions might have been subject to systematic measurement error. Future research could use 24-h recall and food records to measure total intakes of different food components as well as clinical tools for physical activity measurement (accelerometers, pedometers and activity diaries) for more precise assessment of energy expenditure. Second, the fitted models did not control for parental income. Therefore, the reported associations between children’s behaviours and weight status might be confounded by income effects for unhealthy food and sedentary lifestyles. Third, the studied indicators were highly correlated. Dissection of specific influences requires experimental evidence. Fourth, the sample might lack the statistical power to detect the association of children’s diet and physical activity and weight status. Future research might address the issue by leveraging panel data and/or by employing more precise measures of risk factors than self-reported data. Fifth, the sample was relatively homogeneous in terms of demographic characteristics compared with the rest of the country. Six and related, although the previous study based on the same sample^(26)^ reported that the sample characteristics were well aligned with the national census, the classes in schools were not selected at random and that might have affected the representativity of the data. Notwithstanding these limitations, the findings provide useful evidence that could inform future research and family-based interventions to reduce the risk of childhood overweight and obesity in Indonesia.

## Conclusion

This study illustrates the potential association between demographic, behavioural, and environmental risk factors and overweight or obesity among primary schoolchildren in a middle-income country setting. This adds to the growing literature on the prevalence of health risk factors, their coexistence and associations with childhood overweight and obesity in LMIC. To the best of the authors’ knowledge, this is the first attempt to assess the complex relationship between various risk factors and their association with children’s weight across the entire BMI-for-age Z-score distribution in urban Indonesia. This knowledge is required to inform future research and to develop effective interventions and policies to counter the rising prevalence of childhood overweight and obesity in LMIC. Parents of primary schoolchildren can encourage healthy behaviours in their children by raising their nutrition knowledge and providing a supportive home food environment. Future interventions should be sex-responsive given the documented sex differences, target both parents and children, and aim to improve the food environment at homes and schools and continuously promote healthy diet and adequate physical activity.

## Supplementary material

For supplementary material accompanying this paper visit https://doi.org/10.1017/S1368980023000897. click here to view supplementary material
