# Supplementary material for: Behavioural and environmental risk factors associated with primary schoolchildren’s overweight and obesity in urban Indonesia
Source: Public Health Nutr. 2023 May 4;26(8):1562–75. doi: 10.1017/S1368980023000897 (PMC10410387; doi:10.1017/S1368980023000897)
Supplement: Supplementary file 1 [file S1368980023000897sup001.docx]

**Supplementary Appendix A**

***Evidence before this study: a scoping literature review***

A scoping review of peer-reviewed articles published in English between January 2009 and May 2021 was conducted to identify the existing evidence of factors associated with an increased risk of overweight and obesity among children and adolescents aged 6-18 years old in LMICs (the terms for a systematic search of PubMed are provided in Table SA1). Socio-economic status (family income, parental education, and work status)^(1)^, child’s diet^(2)^, and physical (in)activity patterns^(3)^, parental overweight and obesity, parental practices^(4)^, and environment^(5)^ were found to be important contributors to a child’s overweight status.

Previous observational studies and interventions have provided insight into the potential co-occurrence of risk factors. Evidence from integrated interventions, although scarce in LMICs, points to the most important combinations of diet and physical activity^(6,7)^ together with parental influences^(8)^ and children’s cognitive and behavioral distortions^(9)^. At the same time, a large body of observational research has explored the association of childhood overweight with multiple risk factors and their various combinations (for review see^(10,11,12)^).

Several attempts have been made to systematically assess the prevalence and coexistence of a broad range of behavioral and environmental risk factors among schoolchildren and adolescents using large multinational surveys across several LMICs. The data of two of these are widely used in the literature. First, the International Study of Childhood Obesity, Lifestyle, and the Environment (ISCOLE) was conducted in 2011-2013 among 9-11 years old children living in Europe, Africa, the Americas, South and South-East Asia (India, China) to determine the relationships between childhood overweight, dietary patterns, physical activity, sleep, sedentary behavior, and environmental influences at home, school, and neighborhood levels (see^(13)^ for a summary of key contributions). Second, the Global School-Based Health Survey (GSHS) was conducted among nationally representative samples of 13-17 adolescents in two waves (in 2007 and 2015) in Indonesia^(14)^. Successive research has estimated the prevalence of potential behavioral risk factors, such as consumption of fruits and vegetables, soft drinks, and fast food, physical activity, sedentary behavior, psychological aspects, peer and parental influences, and their clustering^(15,16,17,18,19)^.

The systematic literature search yielded only three quantitative, single-country studies in Indonesia. All of them explored associations between child weight status and socioeconomic correlates, eating behavior (dietary patterns, consumption of sugar-sweetened beverages, fast food, and snacks), physical activity, sedentary behavior, and screen time^(20,21,22)^. One study was conducted in 2013 and explored the risk factors associated with obesity among obese and normal-weight children of 7-12 years old (*n* = 488) in Yogyakarta Province^(20)^. Two other cross-sectional studies targeted adolescents (10/12-18 years old). These studies examined the patterns of sedentary and dietary behaviors associated with overweight and obesity among rural and urban youth in a nationally representative sample (*n* = 155,645) from the Indonesia Basic Health Survey 2013^(22)^, and the prevalence and associated socioeconomic, dietary, and activity risk factors of adolescent (*n* = 2,160) malnutrition in two socioeconomically different districts in 2017^(21)^. Additionally, one qualitative study provided insights into mothers’ motivations to make food choices for their children^(23)^.

***References***

1. Williamson VG, Dilip A, Dillard JR *et al.* (2020) The influence of socioeconomic status on snacking and weight among adolescents: A scoping review. *Nutrients* **12**(1), 167.

2. Liberali R, Kupek E & Assis MAAD (2020) Dietary patterns and childhood obesity risk: a systematic review. *Childhood Obesity* **16**(2), 70-85.

3. Rollo S, Antsygina O & Tremblay MS (2020) The whole day matters: understanding 24-hour movement guideline adherence and relationships with health indicators across the lifespan. *Journal of sport and health science* **9**, 493-510.

4. Lindsay AC, Sitthisongkram S, Greaney ML *et al.* (2017) Non-responsive feeding practices, unhealthy eating behaviors, and risk of child overweight and obesity in Southeast Asia: A systematic review. *International journal of environmental research and public health* **14**(4), 436.

5. Sirasa F, Mitchell LJ, Rigby R *et al.* (2019) Family and community factors shaping the eating behaviour of preschool-aged children in low and middle-income countries: A systematic review of interventions. *Preventive medicine* **129**, 105827.

6. Adom T, De Villiers A, Puoane T *et al.* (2020) School-based interventions targeting nutrition and physical activity, and body weight status of African children: A systematic review. *Nutrients* **12**(1), 95.

7. Anselma M, Chinapaw MJ, Kornet-Van der Aa DA *et al.* (2020) Effectiveness and promising behavior change techniques of interventions targeting energy balance related behaviors in children from lower socioeconomic environments: A systematic review. *PloS one* **15**(9), e0237969.

8. Morgan EH, Schoonees A, Sriram U *et al.* (2020) Caregiver involvement in interventions for improving children's dietary intake and physical activity behaviors. *Cochrane Database of Systematic Reviews* **1**, 1-259.

9. Salam RA, Padhani ZA, Das JK *et al.* (2020) Effects of lifestyle modification interventions to prevent and manage child and adolescent obesity: a systematic review and meta-analysis. *Nutrients* **12**(8), 2208.

10. Aceves-Martins M, Llauradó E, Tarro L *et al.* (2016) Obesity-promoting factors in Mexican children and adolescents: challenges and opportunities. *Global health action* **9**(1), 29625.

11. Al-Jawaldeh A, Taktouk M & Nasreddine L (2020) Food consumption patterns and nutrient intakes of children and adolescents in the Eastern Mediterranean Region: A call for policy action. *Nutrients* **12**(11), 3345.

12. Mistry SK & Puthussery S (2015) Risk factors of overweight and obesity in childhood and adolescence in South Asian countries: a systematic review of the evidence. *Public health* **129**(3), 200-209.

13. Katzmarzyk PT, Chaput JP, Fogelholm M *et al.* (2019) International Study of Childhood Obesity, Lifestyle and the Environment (ISCOLE): contributions to understanding the global obesity epidemic. *Nutrients* **11**(4), 848.

14. WHO (2021) Global School-based Student Health Survey. https://www.who.int/teams/noncommunicable-diseases/surveillance/systems-tools/global-school-based-student-health-survey (accessed November 2022).

15. Caleyachetty R, Echouffo-Tcheugui JB, Tait CA *et al.* (2015) Prevalence of behavioural risk factors for cardiovascular disease in adolescents in low-income and middle-income countries: an individual participant data meta-analysis. *The lancet Diabetes & endocrinology* **3**(7), 535-544.

16. Pengpid S & Peltzer K (2019) Underweight and overweight or obesity and associated factors among school-going adolescents in five ASEAN countries, 2015. *Diabetes & Metabolic Syndrome: Clinical Research & Reviews* **13**(6), 3075-3080.

17. Qi W, Zhou Y, You D *et al.* (2021) Cardiovascular health behaviours of young adolescents: Results from the global school‐based student health survey. *Journal of Paediatrics and Child Health* **57**(4), 566-573.

18. Sultana S, Rahman MM, Sigel B *et al.* (2021) Associations of lifestyle risk factors with overweight or obesity among adolescents: a multicountry analysis. *The American Journal of Clinical Nutrition* **113**(3), 742-750.

19. Uddin R, Lee EY, Khan SR *et al.* (2020) Clustering of lifestyle risk factors for non-communicable diseases in 304,779 adolescents from 89 countries: A global perspective. *Preventive medicine* **131**, 105955.

20. Hadi H, Nurwanti E, Gittelsohn J *et al.* (2020) Improved Understanding of Interactions between Risk Factors for Child Obesity May Lead to Better Designed Prevention Policies and Programs in Indonesia. *Nutrients* **12**(1), 175.

21. Maehara M, Rah JH, Roshita A *et al.* (2019) Patterns and risk factors of double burden of malnutrition among adolescent girls and boys in Indonesia. *PloS one* **14**(8), e0221273.

22. Nurwanti E, Hadi H, Chang JS *et al.* (2019) Rural–urban differences in dietary behavior and obesity: results of the Riskesdas study in 10–18-year-old Indonesian children and adolescents. *Nutrients* **11**(11), 2813.

23. Rachmi CN, Hunter CL, Li M *et al.* (2018) Food choices made by primary carers (mothers/grandmothers) in West Java, Indonesia. *Appetite* **130**, 84-92.

**Table SA1** Search terms for systematic literature review

| Database | Set | Category | Terms |
| --- | --- | --- | --- |
| PubMed | 1 | Population | (child* OR adolescen* OR youth* OR young people OR young person* OR school* OR schoolchild* OR school child* OR boys* OR girls*) |
|  | 2 | Factor | (home food environment OR food environment OR obesogenic environment OR obesogenic home OR unhealthy home OR family meal* OR family mealtime* OR family meal-time* OR family dinner* OR food availability OR home food OR available food OR food access* OR food in home OR parental monitoring OR parental modeling OR parental policies OR parental role model OR parental knowledge OR home activity OR home physical activity OR sedentary behavior OR sedentary behaviour OR home media OR home media environment OR tv viewing OR screen time* OR screen-time OR media physical environment OR media social environment OR parental activity OR family physical activity OR physical activity OR social environment OR physical home environment) |
|  | 3 | Behavior | (snacking OR eating behavior* OR eating behaviour OR food intake* OR fruit* OR fruit* consum* OR fruit* intake* OR vegetable* OR vegetable* consum* OR vegetable* intake* OR fruit and vegetable OR eat* OR diet* OR calori* OR energy OR meal OR menu OR nutrition*) |
|  | 4 | Outcome | (obesity OR overweight OR weight OR body weight OR weight status OR bmi OR body mass index OR anthropometr* OR BMI z-scores or BMI-for-age OR weight-for-length OR waist circumference OR adipos*) |
|  | 5 | Setting | (developing countr* OR low income * OR middle income * OR low* and middle* income countr*) |

*Notes:* The systematic search of PubMed was conducted in May 2021and was limited to articles published between January 2009 and April 2021. An asterisk (*) is added to the search terms as a placeholder for any unknown or wildcard term. Terms within sets were connected using OR, and terms between sets were connected using AND.

**Supplementary Appendix B**


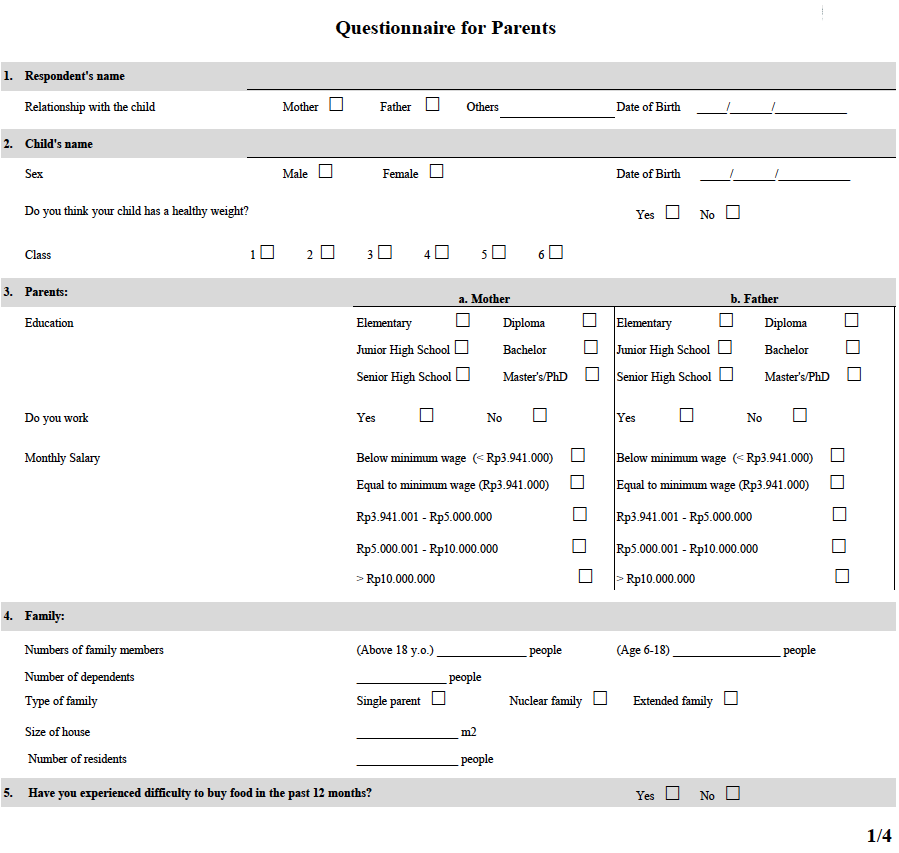


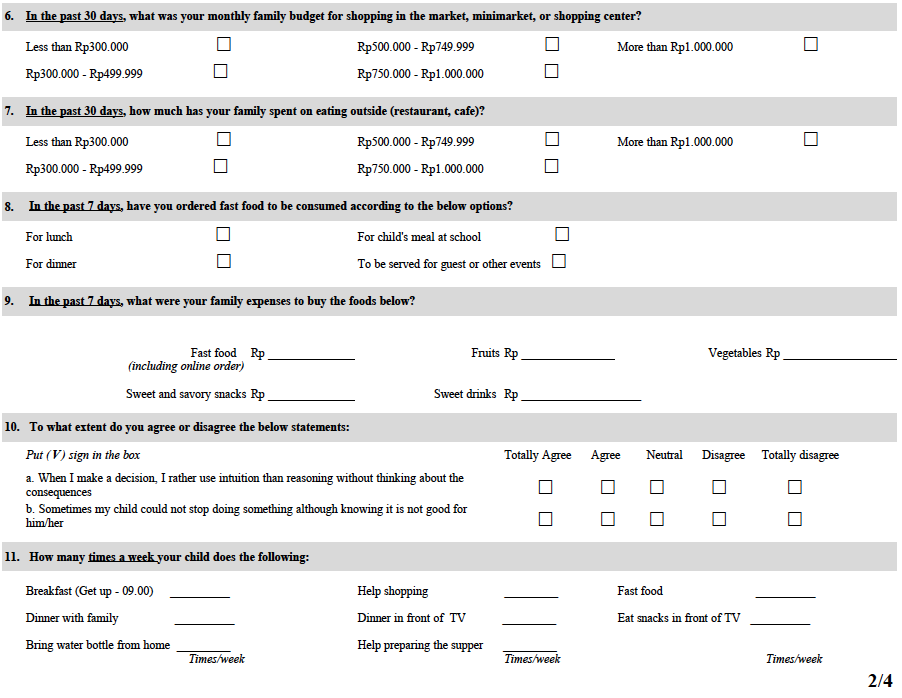


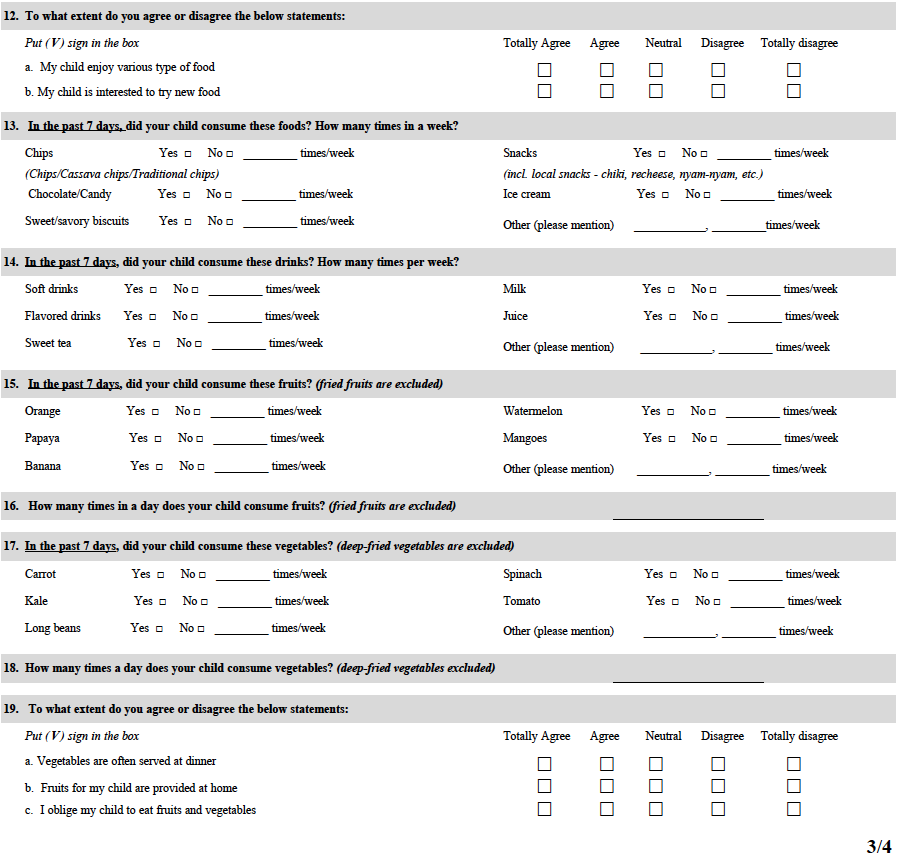


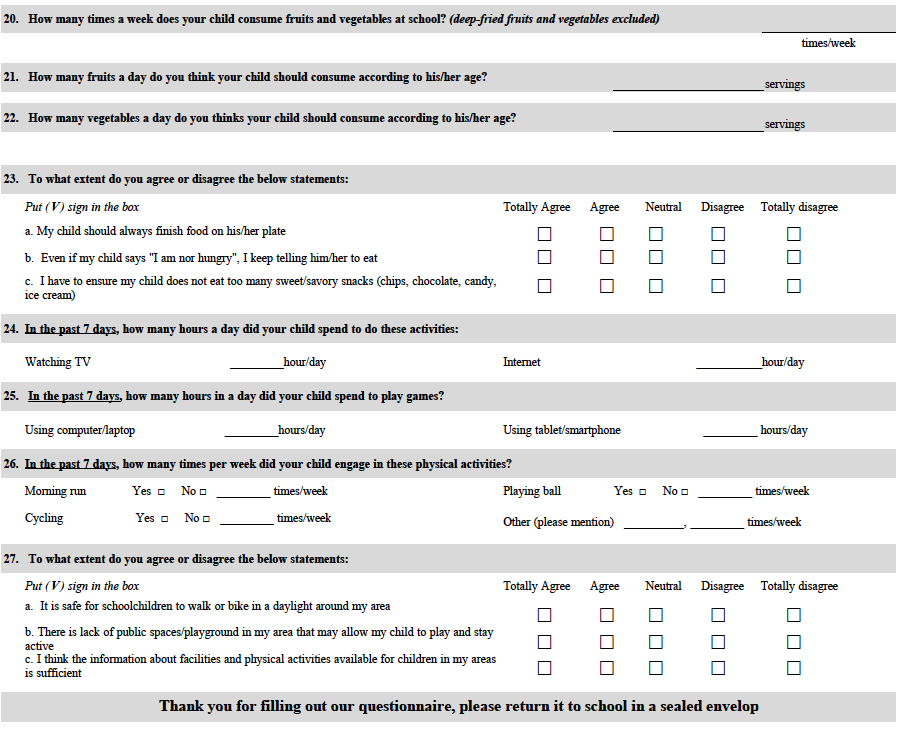


**
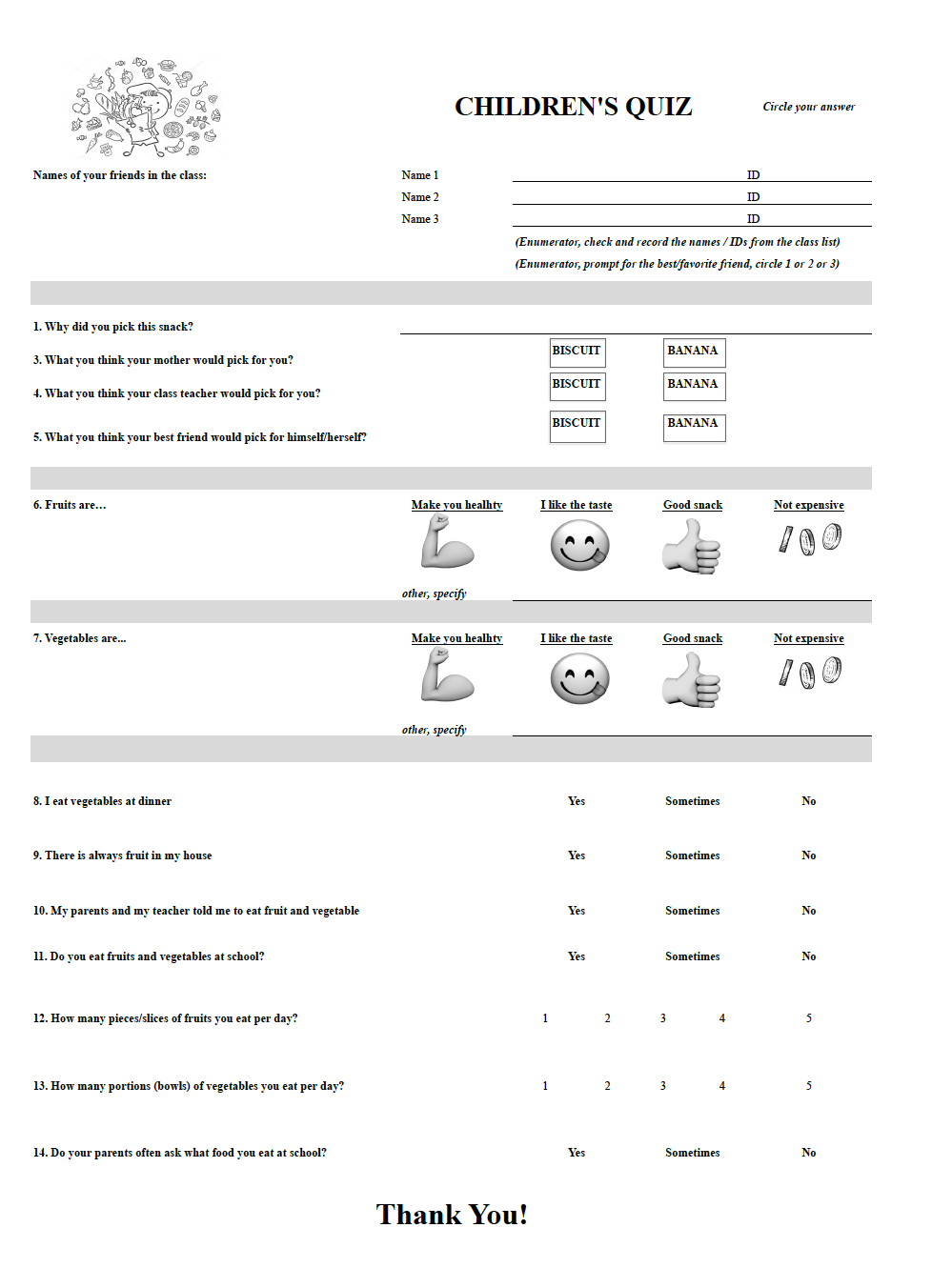
**

**Supplementary Appendix C**

**Table SC1** Risk factors of overweight and obesity among children in selected primary schools in Jakarta

|  |  | (1) | (2) | (3) | (4) |
| --- | --- | --- | --- | --- | --- |
| Child is male |  | 0.090*** | 0.089*** | 0.090*** | 0.083*** |
|  |  | (0.022) | (0.021) | (0.022) | (0.021) |
| Child's age, years |  | -0.147*** | -0.153*** | -0.148*** | -0.154*** |
|  |  | (0.012) | (0.011) | (0.012) | (0.011) |
| Child's height, cm |  | 0.026*** | 0.027*** | 0.026*** | 0.027*** |
|  |  | (0.001) | (0.001) | (0.001) | (0.001) |
| Mother's years of education | | 0.004 | 0.005 | 0.004 | 0.005 |
|  |  | (0.004) | (0.004) | (0.004) | (0.004) |
| Child's dietary risk score (0-5) | | 0.008 | 0.014 |  |  |
|  |  | (0.010) | (0.009) |  |  |
| Child's physical activity risk score (0-2) | | 0.008 |  | 0.009 |  |
|  |  | (0.020) |  | (0.020) |  |
| Obesogenic home food environm. score (0-6) | | 0.017 |  |  | 0.017* |
|  |  | (0.009) |  |  | (0.008) |
| Constant |  | -1.997*** | -2.002*** | -1.920*** | -2.026*** |
|  |  | (0.157) | (0.146) | (0.153) | (0.148) |
| Number of observations | | 1,538 | 1,628 | 1,547 | 1,631 |

*Abbreviation:* d, dummy variable; environm., environment.

*Notes:* Linear probability model, robust standard errors. **Dependent variable**: A binary overweight indicator based on the BMI-for-age Z-score above +1 standard deviation according to the WHO Child Growth Standards. All specifications include a dummy for missing observations of age and maternal years of education, which were interpolated with the sample mean. ** P*<0.05, ** *P*<0.01, *** *P*<0.001.

**Table SC2** Risk factors of overweight and obesity among children in selected primary schools in Jakarta

|  |  | (1) | | (2) | | (3) | | (4) | |
| --- | --- | --- | --- | --- | --- | --- | --- | --- | --- |
|  |  | aOR | 95% CI | aOR | 95% CI | aOR | 95% CI | aOR | 95% CI |
|  |  |  |  |  |  |  |  |  |  |
| Child is male, *d* | | 1.66*** | [1.29, 2.14] | 1.64*** | [1.29, 2.09] | 1.66*** | [1.29, 2.13] | 1.59*** | [1.25, 2.02] |
|  |  |  |  |  |  |  |  |  |  |
| Child's age, years | | 0.43*** | [0.37, 0.50] | 0.41*** | [0.35, 0.48] | 0.43*** | [0.37, 0.51] | 0.41*** | [0.35, 0.48] |
|  |  |  |  |  |  |  |  |  |  |
| Child's height, cm | | 1.16*** | [1.14, 1.19] | 1.17*** | [1.14, 1.19] | 1.16*** | [1.14, 1.19] | 1.17*** | [1.15, 1.20] |
|  |  |  |  |  |  |  |  |  |  |
| Mother's years of education | | 1.03 | [0.98, 1.08] | 1.03 | [0.98, 1.08] | 1.02 | [0.98, 1.07] | 1.03 | [0.98, 1.08] |
|  |  |  |  |  |  |  |  |  |  |
| Child's dietary risk score (0-5) | | 1.05 | [0.93, 1.18] | 1.09 | [0.98, 1.22] |  |  |  |  |
|  |  |  |  |  |  |  |  |  |  |
| Child's physical activity risk score (0-2) | | 1.06 | [0.85, 1.33] |  |  | 1.07 | [0.86, 1.34] |  |  |
|  |  |  |  |  |  |  |  |  |  |
| Obesogenic home food environm. score (0-6) | | 1.10 | [1.00, 1.22] |  |  |  |  | 1.11* | [1.01, 1.22] |
|  |  |  |  |  |  |  |  |  |  |
| School controls | | Yes | | Yes | | Yes | | Yes | |
|  |  |  |  |  |  |  |  |  |  |
| Number of observations | | 1,538 | | 1,628 | | 1,547 | | 1,631 | |

*Abbreviation:* d, dummy variable; environm., environment.

*Notes:* Logistic regression model. Robust standard errors. **Dependent variable**: A binary overweight indicator based on the BMI-for-age Z-score above +1 standard deviation according to the WHO Child Growth Standards. All specifications included a dummy for missing observations of age and maternal years of education, which were interpolated with the sample mean; and 17 school dummy variables. ** P*<0.05, ** *P*<0.01, *** *P*<0.001.
